# What proportion of care home outbreaks are caused by norovirus? An analysis of viral causes of gastroenteritis outbreaks in care homes, North East England, 2016–2018

**DOI:** 10.1186/s12879-019-4726-4

**Published:** 2019-12-31

**Authors:** Thomas Inns, Deb Wilson, Petra Manley, John P. Harris, Sarah J. O’Brien, Roberto Vivancos

**Affiliations:** 1 Field Epidemiology, Field Service, National Infection Service, Public Health England, London, UK; 2 Institute of Population Health Sciences, University of Liverpool, Liverpool, UK; 3 NIHR Health Protection Research Unit in Gastrointestinal Infections, University of Liverpool, Liverpool, UK; 4 North East Health Protection Team, Public Health England, Newcastle-upon-Tyne, UK; 5 NIHR Health Protection Research Unit in Emerging and Zoonotic Infections, University of Liverpool, Liverpool, UK

**Keywords:** Norovirus, Gastroenteritis, Outbreaks, Surveillance

## Abstract

**Background:**

Outbreaks of infectious gastroenteritis are common in care homes for the elderly. Norovirus can cause these outbreaks, but diagnosis is frequently based solely on clinical characteristics. Our objective in this study was to describe the epidemiology of norovirus and other gastrointestinal pathogens in these settings.

**Methods:**

We analysed surveillance data from gastroenteritis outbreaks reported in North East England between 04 July 2016 to 01 July 2018. Stool samples taken during these outbreaks were tested for a range of viral and bacterial pathogens. We described the epidemiology of these outbreaks and explored the characteristics of norovirus outbreaks versus from other viral causes using multivariable logistic regression.

**Results:**

From the 566 care home gastroenteritis outbreaks in this study, we found that norovirus was the pathogen most frequently isolated. Norovirus was detected in 64% of outbreaks with a pathogen identified. Sapovirus was found in 13%; rotavirus in 11%. We found that norovirus outbreaks were associated with higher attack rates (aOR 1.03, 95% CI 1.01–1.05) and fewer cases sampled (aOR 0.74, 95% CI 0.60–0.91), compared to outbreaks caused by other viral pathogens.

**Conclusions:**

These results are important as they quantify the contribution of norovirus to gastroenteritis outbreaks in care homes. Given this evidence, we emphasize the importance of non-specific outbreak interventions that can affect the impact of all such outbreaks. We further recommend that these findings are used to inform the implementation strategies of any norovirus-specific interventions such as a norovirus vaccine.

## Background

Acute infectious gastroenteritis is a common cause of morbidity in the general population [1]. Residential care homes for the elderly (also known as long-term care facilities), including those offering nursing care, provide an environment suited to the acquisition and spread of infectious agents causing gastroenteritis [2]. Because of this, outbreaks of acute gastroenteritis in semi-enclosed settings such as care homes are difficult to prevent and challenging to control [3]. This is a public health concern because the morbidity and mortality associated with gastroenteritis outbreaks is higher amongst the elderly residents of care homes [4, 5].

Norovirus has been reported as the most frequent cause of care home gastroenteritis outbreaks [6]. Norovirus outbreaks are particularly difficult to prevent in care homes due to its low infectious dose, the lack of long term immunity to reinfection, its persistence in the environment and the possibility of infected persons shedding virus asymptomatically [7]. There are other viral pathogens such as sapovirus, astrovirus, rotavirus and adenovirus which have been reported to have caused outbreaks of gastroenteritis in care homes [8, 9]. However, there is limited evidence-base to understand the relative contribution of these viral pathogens, and bacterial pathogens such as *Salmonella* and *Campylobacter*, to the total burden of care home gastroenteritis outbreaks in England. Despite this lack of evidence, outbreaks of diarrhoea and vomiting in care homes are commonly classed as being caused by norovirus, on clinical and epidemiological characteristics alone, which may lead to a substantial overestimate of the burden of norovirus outbreaks [10].

The aim of this study was to describe the epidemiology of gastroenteritis outbreaks in care homes in North East England, with particular reference to norovirus.

## Methods

### Setting

In England, all care homes (residential facilities providing social and nursing care to the elderly) are required to be registered with and inspected by the Care Quality Commission (CQC) in accordance with Schedule 1 of The Health and Social Care Act 2008 (Regulated Activities) Regulations 2014. Care homes are advised by the CQC to report outbreaks to Public Health England (PHE) but this is not mandatory [11]. In North East England, there are 12 local authority areas with a total population of 2.645 million in 2017, and 742 CQC registered care homes. PHE North East operates a surveillance system for gastroenteritis outbreaks in care homes. The study population comprised all North East CQC registered care homes. The study included all gastroenteritis outbreaks reported from 04 July 2016 to 01 July 2018.

### Outbreak definition

An outbreak was defined as two or more cases of diarrhoea and/or vomiting occurring in staff and/or residents in the same home within a short time period [12]. No standardised definitions were used for “diarrhoea” or “a short period of time”. The start of an outbreak was defined as the date of onset in the index case; the end of an outbreak was defined as 72 h after the resolution of symptoms in the last case. Outbreak reports were received by PHE and entered on to an electronic case management system (HPZone) [13].

### Pathogen detection

All study care homes were asked to submit stool samples from at least six cases during outbreaks of infectious gastroenteritis. All these stool samples were processed in one laboratory in Newcastle-upon-Tyne, tested for the following pathogens; *Campylobacter* sp., *Salmonella* sp., *E. coli* O157, *Shigella* sp. (all using culture), norovirus, sapovirus, rotavirus, astrovirus, adenovirus (all using multiplex PCR), *C. difficile* (three stage testing following national guidance) [14] and *Cryptosporidium* spp.(Phenol-auramine staining and fluorescence microscopy). Stools samples were only tested for *C. perfringens* and *B. cereus* if this was requested based on clinical and epidemiological assessment of the outbreak. All pathogen testing was conducted using agreed laboratory standards [15]. Laboratory results were recorded daily on a Structured Query Language (SQL) database.

### Data collection

Epidemiological data captured in the surveillance system included variables such as: number of residents and staff at the home, the number of cases in residents and staff, date of outbreak onset and outbreak duration. Laboratory data included number of stool samples, number of cases tested and pathogen testing results. Data were extracted from HPZone, with data checked against paper outbreak records. Laboratory data were extracted from the relevant SQL database and joined with the epidemiological data using a unique outbreak identifier.

### Data analysis

We calculated the incidence rate of care home gastroenteritis outbreaks per 100 care homes per year for each local authority and the percentage of outbreaks with stool samples submitted. We calculated resident attack rates as number of cases divided by number of residents. The number of residents and duration of outbreak were included as continuous variables. We calculated the ratio of staff to residents and used this as a continuous variable. Outbreaks with an onset date after week 42 and before week 16 (based on ISO 8601) were classed as occurring during winter and analysed as a binary variable. We described the number of outbreaks by month of onset. We used loess regression to fit smooth curves to show the change in the percentage of outbreaks with a sample submitted over time and the change in the percentage of outbreaks norovirus positive over time. Where only one pathogen was isolated from an outbreak, this was assigned as the cause. We also described outbreaks where more than one pathogen was identified.

We used those outbreaks with a single viral pathogen identified for a multivariable analysis. The outcome was detection of norovirus. Outbreaks with no pathogen or multiple pathogens identified were excluded from the multivariable analysis. We used a mixed-effects logistic regression model to explore the characteristics of norovirus infection versus other viral causes, simultaneously adjusted for all other explanatory variables. Random care home-level intercepts were used to account for within-home correlation. The explanatory variables included a priori as we believed them to be associated with norovirus outbreaks were: resident attack rate, care home population, outbreak duration and number of cases sampled. The other three variables (number of virus-positive samples, ratio of staff to residents and winter) were regarded as potential confounders so added to the model and retained if they improved model fit, as measured using the Akaike information criterion (AIC) [16]. Interaction terms between parameters were added and tested for significance using a likelihood-ratio test from the lmtest package and then assessed using AIC for improved model fit if significant [17]. All analyses were conducted using R 3.5.0 [18].

## Results

During the study period we recorded a total of 566 outbreaks from 339 care homes. This equates to an incidence rate of 38.14 outbreaks per 100 homes per year. Of the 339 care homes reporting outbreaks, 194 (57.2%) reported only one outbreak during the study period, with the maximum being 7 outbreaks reported by one care home. Of the 566 outbreaks, at least one stool sample was submitted for laboratory testing for 362 (64.0%) outbreaks.

A breakdown of the number of care homes, number and incidence rate of outbreaks, and number and percentage of outbreaks with a faecal sample submitted for pathogen testing is shown in Table 1. The area with the lowest incidence rate of outbreaks was Redcar and Cleveland (25.5 outbreaks per 100 care homes per year) and the highest was North Tyneside (64.1 outbreaks per 100 care homes per year). The area with the highest percentage of outbreaks with a stool sample submitted was County Durham (72%), which was substantially higher than the percentage from Newcastle upon Tyne (51.3%), the lowest of the 12 areas.

**Table 1:** Care home gastroenteritis outbreaks by local authority area (*n* = 566), North East England, 2016–2018

Local Authority	Total registered care homes	Home reporting an outbreak	Percentage of homes with outbreak	Outbreaks	Outbreaks per 100 care homes per year	Outbreaks with samples submitted	Percentage with samples submitted
County Durham	144	66	45.8	107	37.2	79	72.0
Darlington	33	18	54.5	32	48.5	21	65.6
Gateshead	66	30	45.5	55	41.7	35	60.0
Hartlepool	23	15	65.2	20	43.5	11	55.0
Middlesbrough	43	17	39.5	27	31.4	19	70.4
Newcastle upon Tyne	62	28	45.2	39	31.5	20	51.3
North Tyneside	46	30	65.2	59	64.1	33	54.2
Northumberland	98	45	45.9	75	38.3	49	64.0
Redcar and Cleveland	53	15	28.3	27	25.5	19	66.7
South Tyneside	32	18	56.2	29	45.3	20	65.5
Stockton-on-Tees	53	22	41.5	36	34.0	21	58.3
Sunderland	89	35	39.3	60	33.7	35	58.3
Total	742	339	45.7	566	38.1	362	64.0

**Fig. 1:**
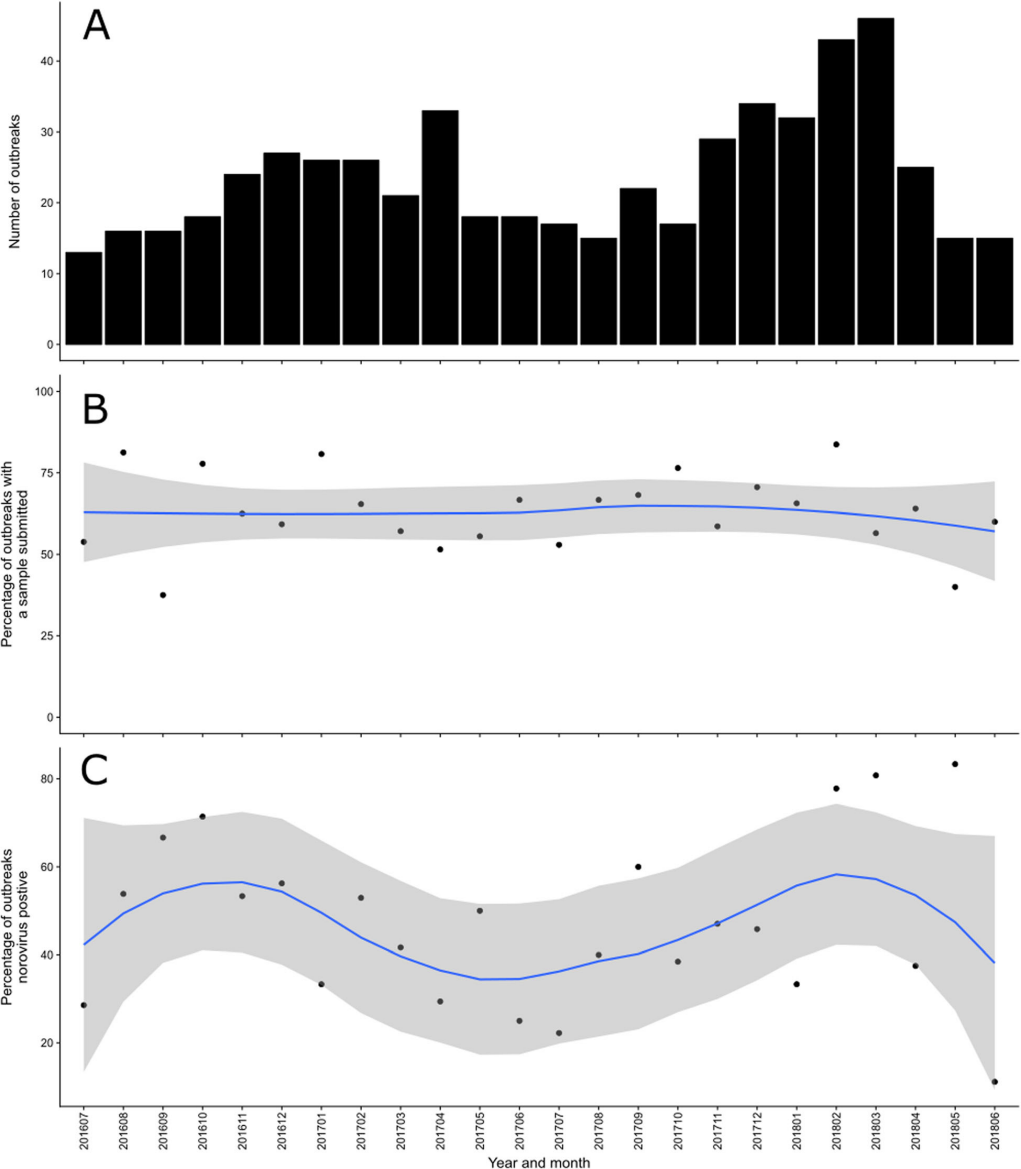
Care home gastroenteritis outbreaks by month and year, North East England, 2016–2018. **a** Total number of outbreaks. **b** Percentage of outbreaks with a faecal sample submitted, with loess regression smoothed line (blue line) and 95% Confidence Interval (grey). **c** Percentage of outbreaks with a positive norovirus sample, with loess regression smoothed line (blue line) and 95% Confidence Interval (grey)

The temporal distribution of outbreaks is shown in Fig. 1a. In the 2016/17 season the month with the largest number of outbreaks was April 2017 (*n* = 33). During the 2017/18 season there were more outbreaks than the previous season, with the number of outbreaks peaking in March 2018 (*n* = 46). The percentage of outbreaks with a sample submitted is shown over time in Fig. 1b. There was variation in the percentage submitted by month, with the lowest in September 2016 (37.5%) and highest in February 2018 (83.7%), however there was no notable trend or periodicity.

From the 362 laboratory-tested outbreaks, a pathogen was detected in 284 (78.5%) outbreaks; of these 263 (92.6%) had a viral pathogen identified, 257 (90.4%) with a single viral cause. Six viruses were detected, in order of frequency: 181 norovirus (64%), 37 sapovirus (13%), 32 rotavirus (11%), six astrovirus (2%) and one adenovirus (0.4%). Norovirus and sapovirus were detected together in 2 outbreaks; norovirus and rotavirus were also detected together in 2 outbreaks. *Clostridium difficile* was detected in 4 outbreaks and *Campylobacter* in 2 outbreaks. Norovirus and *C. difficile* were detected together in 2 outbreaks. There were only 38 outbreaks for which samples were tested for *C. perfringens* and *B. cereus*. *C. perfringens* was identified in 15 (39%) of these outbreaks, but the toxin gene was only detected in three outbreaks. *B. cereus* was not identified in any outbreaks.

Overall, 50% of the 362 outbreaks with a sample submitted had a positive norovirus result and no other pathogen detected. The percentage of outbreaks with a sample submitted that was positive for norovirus is shown by month in Fig. 1c. Norovirus was detected in every month, with the lowest proportion of outbreaks being caused by norovirus in June 2018 (11%). There was a seasonal change in this relationship, with a higher percentage of samples positive for norovirus during the winter months in both seasons.

**Fig. 2:**
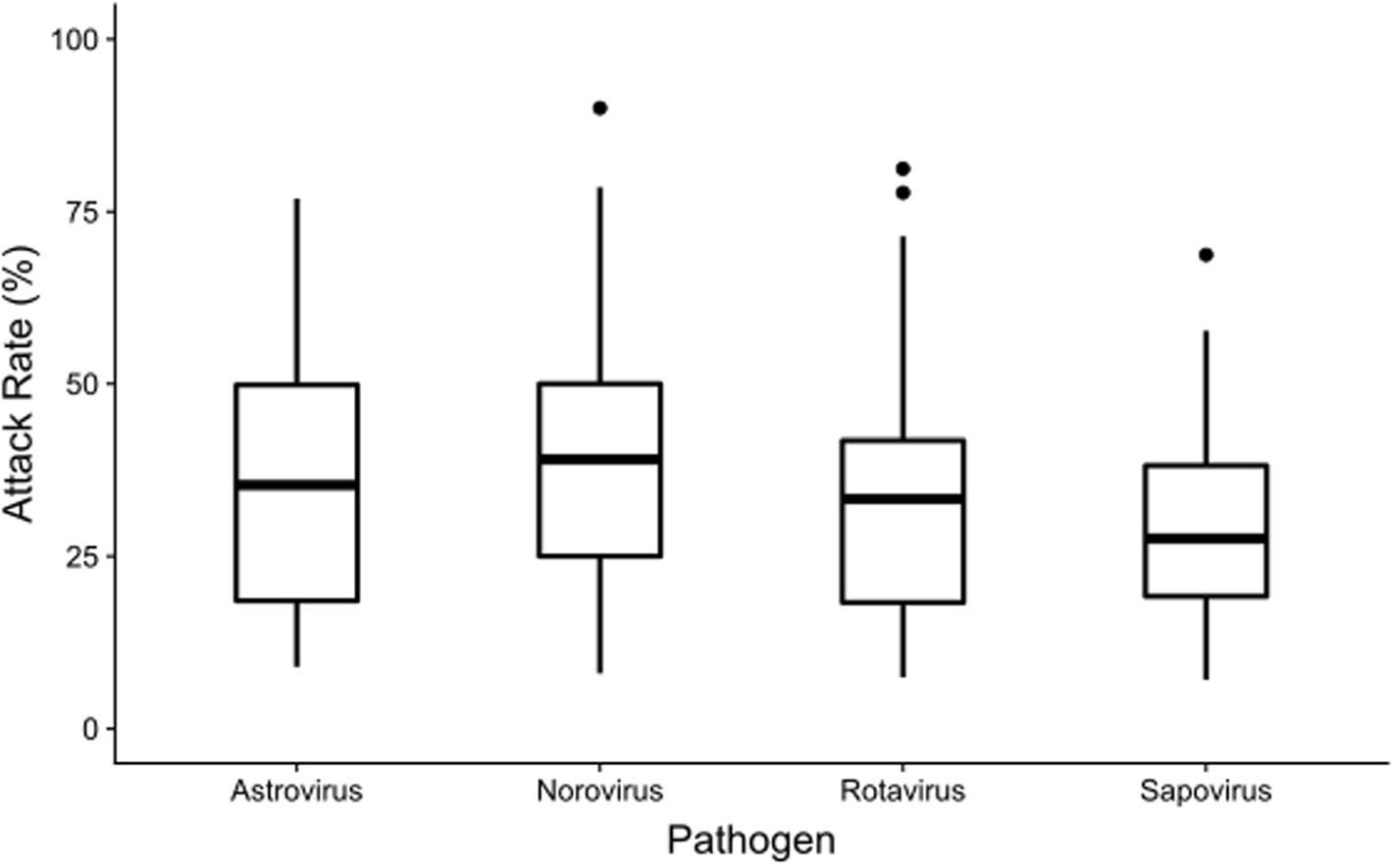
Boxplot showing the distribution of attack rates in residents during care home gastroenteritis outbreaks of astrovirus, norovirus, rotavirus and sapovirus (*n* = 256*), North East England, 2016–2018. *excluding one adenovirus outbreak

The median population (residents and staff) of care homes in this dataset was 96 people (Interquartile range (IQR) 70–121); the median number of residents was 44 (IQR 34–57). The median ratio of staff to residents was 1.16:1 (IQR 0.99:1–1.38:1). Of the 284 outbreaks where a stool sample was submitted, the median number of cases tested was 3 (IQR 2–4). The median attack rate in residents was 27.3% (IQR 15.7–41.7%). For those 256 outbreaks of astrovirus, norovirus, rotavirus and sapovirus the distribution of resident attack rates is shown by pathogen in Fig. 2: this excludes the one adenovirus outbreak. The attack rate was highest in norovirus outbreaks (39.1%), followed by astrovirus outbreaks (35.4%), rotavirus outbreaks (33.3%) and sapovirus outbreaks (27.6%). However, these differences in AR are not statistically significant (Fig. 2).

For the 257 outbreaks of a single viral cause, the association between various outbreak characteristics and norovirus detection is shown in Table 2. These are compared with outbreaks where sapovirus, rotavirus, astrovirus or adenovirus were identified. In the univariable analysis norovirus outbreaks had a significantly higher attack rate; Odds Ratio (OR) 1.02 (95% CI 1.01–1.05). None of the other outbreak characteristics such as care home population size, outbreak duration, number of cases tested, number of virus-positive samples, outbreak during winter or the staff to resident ratio were significantly associated with norovirus in the univariable analysis. In the multivariable analysis, when simultaneously adjusted for other variables, higher attack rates in residents were significantly associated with norovirus (aOR 1.03, 95%CI 1.01–1.05). Norovirus was also significantly associated with fewer cases being sampled (aOR 0.74, 95% CI 0.60–0.91). No other variables were significantly associated with norovirus outbreaks in the multivariable model. No interaction terms were statistically significant.

**Table 2:** Association between outbreak characteristics and norovirus detection, care home gastroenteritis outbreaks of a confirmed viral cause (*n* = 257), North East England, 2016–2018

Variable	Other viruses (*n* = 76)		Norovirus (*n* = 181)		OR	*p* value	aOR	95% Confidence Interval		*p* value
	Mean	SD	Mean	SD						
Resident attack rate	33.04	*19.27*	38.30	*17.74*	1.02	0.038	1.03	1.01	1.05	0.004
Number of residents	44.75	*19.58*	45.73	*16.41*	1.00	0.679	1.02	0.99	1.04	0.077
Outbreak duration	20.84	*10.41*	18.87	*10.60*	0.98	0.174	0.98	0.95	1.01	0.130
Number of cases sampled	3.66	*2.33*	3.17	*1.83*	0.89	0.080	0.74	0.60	0.91	0.005
Number of virus-positive samples	2.46	*1.71*	2.53	*1.55*	1.03	0.749	1.25	0.97	1.62	0.091
Outbreak in winter? (number and percentage	48	*63.20*	129	*71.30*	1.45	0.201	.	.	.	.
Ratio of staff to residents	1.21	*0.38*	1.21	*0.40*	1.02	0.965	1.17	0.53	2.57	0.701

## Discussion

In this study, we found that norovirus was the pathogen most frequently isolated during care home gastroenteritis outbreaks. Norovirus was the single pathogen identified for 64% of outbreaks where a pathogen was identified; from this we infer that norovirus was the primary cause of gastroenteritis outbreaks in care homes. The percentage of norovirus outbreaks was broadly consistent during the year, but there was some evidence that norovirus accounted for a greater percentage of outbreaks during winter months. We found norovirus outbreaks to be associated with higher attack rates and fewer cases being sampled. Regarding the association between norovirus and higher attack rates, a recent systematic review found that attack rates were influenced by resident mobility and dependency, staff–resident contact intensity, exposure to vomit, and route of feeding [19]. However it was not possible to assess these relationships in this study as these data were not collected.

In this setting the proportion of stool samples submitted for pathogen testing (64%) was slightly higher than the rate of stool testing during care home gastroenteritis outbreaks in other settings in England [20] and France [21]. Given the high proportion of outbreaks tested for a range of pathogens, we have greater assurance in our findings regarding the relative importance of different pathogens in care home gastroenteritis outbreaks. However, the median number of cases sampled was three, which is lower than the recommended guideline of at least six cases, reducing the likelihood of pathogen detection. There remains a possibility that outbreaks where a sample had been submitted were systematically different from those where no sample was submitted, leading to some bias within these results.

In this study, we found that 92.6% of outbreaks with a pathogen detected had a viral pathogen identified. This proportion of outbreaks with a viral cause is substantially higher than a previous study in England and Wales between 1992 and 1994 which attributed 57% of outbreaks in residential facilities where a sample was submitted to viral causes and 29% to bacterial causes, but this may be due to improvements in *Salmonella enteritidis* control in United Kingdom (UK) eggs and in viral detection methodologies [22]. A similar picture was seen in systematic review of such outbreaks published between January 1997 to June 2007, which assigned 69% to viral causes and 31% to bacterial causes [23]. Our finding that a greater proportion of outbreaks had a viral cause may reflect changes in: the completeness of reporting, the food hygiene arrangements, the probability of bias introduced by voluntary sampling, infection control practices and inter-annual changes in viral circulation.

One of the strengths of this study was that all outbreak samples were tested for a wide range of viral and bacterial causes, giving us confidence in the pathogen results produced. After norovirus, the next most frequently identified pathogen in this study was sapovirus, attributed as causing 13% of outbreaks. Sapovirus was the second most common viral pathogen identified in a large community study in the UK [24] and has previously been identified in 66% of norovirus-negative care home outbreak samples in one study in the US [9]. However, sapovirus was not detected in one study of care home outbreaks in The Netherlands [8] and was not tested for during several other studies in similar populations [25, 26]. Similarly, rotavirus was detected in 11% of our study outbreaks. This is consistent with findings from other studies that it can cause a gastroenteritis outbreaks in this population [8, 25] and a study in France which found that norovirus and rotavirus together accounted for 95% of gastroenteritis outbreaks in care homes [21]. Our finding that rotavirus was the third most frequently observed pathogen demonstrated the continued circulation of this virus in the elderly, despite the introduction of rotavirus vaccine for infants in the UK in 2013, and the corresponding decrease in the total number of cases, primarily in infants and toddlers [27].

The results of this study were obtained from one comparatively small area of England over a two-year period. We recognise that as such, there is a question as to whether these findings are representative of the situation over time and in other areas of England. However, in 2016/17 and 2017/18 the number of norovirus laboratory reports in England and Wales were comparable to those seen in the previous 3 years [28], indicating that the national burden of norovirus was similar to previous years and therefore comparable. Although the study took place in one contiguous geographical area, a recent analysis of care home gastroenteritis outbreaks in England show that the rates in this area were equivalent to other areas in England [29]. Given this, we believe that it would be reasonable to generalise these study results to other seasons and other areas of England. As to generalisation to other countries, the appropriateness of this would depend on factors such as: the levels of pathogens circulating at the time of surveillance, different residential populations, different organisational or structural settings, and different infection control practices.

In this study one of the possible limitations was the definition used for attributing a causal pathogen to an outbreak, where we assigned a pathogen as causal if only that pathogen was identified in that outbreak. This is a point of difference from other studies in similar settings [25] which have used previous Centers for Disease Control and Prevention (CDC) definitions that require two or more stool samples with an aetiological agent to assign it as a cause [30]. One effect of using our study definition may be that it is less specific and incorrectly allocates outbreaks not caused by that pathogen. However, we believe our approach is justified as it is consistent with the more recent surveillance definition for norovirus gastroenteritis used by CDC for long-term care facilities [31].

Another possible limitation was that we did not include staff in the care home population size or attack rate. We made this decision due to our concern of under- or over-reporting illness in care home staff. This could have biased our findings relating to attack rates, as the attack rate may have been reduced/inflated for those homes with a higher proportion of staff. We did however include staffing levels with our analysis by including the ratio of residents to staff in the home.

Comparing these results in the context of international literature is difficult due to the different methods of surveillance for outbreaks, varying sampling regimes and the range of different pathogens tested for using a range of methodologies. However, our findings were broadly consistent with other studies which found similar percentage of norovirus outbreaks in other settings such as: Oregon (77%) [32], the Netherlands (78%) [8] and south west England (74%) [26]. Comprehensive surveillance of such outbreaks in Australia (40%) [25] and France (36%) [21], found lower percentages attributed to norovirus, although a direct comparison is difficult ask Kirk et al. defined an outbreak as being caused by norovirus only if at least two samples were positive. The percentage of care home outbreaks attributed to norovirus in our study is substantially higher than the estimated prevalence of norovirus found in sporadic cases of acute gastroenteritis in the community in a worldwide meta-analysis (24%) [33]. This may reflect the increased susceptibility of care home residents [6], the opportunities for transmission [2] and the difficulty of implementing effective infection control measures in such a setting [23].

## Conclusions

In this study, we quantified the percentage of care home gastroenteritis outbreaks attributed to norovirus and other pathogens. We found that norovirus caused 64% of outbreaks where a pathogen was identified and that further 27% of care home outbreaks were caused by a different viral pathogen. Given this evidence, we emphasize the importance of non-specific outbreak interventions such as good hygiene, prompt reporting and strong infection control procedures that can affect the impact of all such outbreaks. However, norovirus-specific interventions (such as a norovirus vaccine) could prevent up to two thirds of outbreaks, that are associated with the highest attack rates.

## Data Availability

The datasets used and analysed during this study are available from the corresponding author on reasonable request.

## Abbreviations

AIC: Akaike Information Criterion
aOR: Adjusted Odds Ratio
AR: Attack Rate
CDC: Centers for Disease Control and Prevention
CI: Confidence Interval
CQC: Care Quality Commission
IQR: Interquartile range
OR: Odds Ratio
PCR: Polymerase Chain Reaction
PHE: Public Health England
SD: Standard Deviation
SQL: Structured Query Language

## References

[1] Walker CLF, Black RE (2010). Diarrhoea morbidity and mortality in older children, adolescents, and adults. Epidemiol Infect.

[2] Strausbaugh LJ, Sukumar SR, Joseph CL (2003). Infectious disease outbreaks in nursing homes: an unappreciated hazard for frail elderly persons. Clin Infect Dis.

[3] Harris JP, Lopman BA, O'Brien SJ (2010). Infection control measures for norovirus: a systematic review of outbreaks in semi-enclosed settings. J Hosp Infect.

[4] Gustavsson L, Andersson L-M, Lindh M, Westin J (2011). Excess mortality following community-onset norovirus enteritis in the elderly. J Hosp Infect.

[5] Trivedi TK, DeSalvo T, Lee L (2012). Hospitalizations and mortality associated with norovirus outbreaks in nursing homes, 2009-2010. JAMA.

[6] Chen Y, Hall AJ, Kirk MD (2017). Norovirus disease in older adults living in long-term care facilities: strategies for management. Curr Geriatr Rep.

[7] Costantini VP, Cooper EM, Hardaker HL, Lee LE, Bierhoff M, Biggs C, Cieslak PR, Hall AJ, Vinje J (2016). Epidemiologic, Virologic, and host genetic factors of Norovirus outbreaks in long-term care facilities. Clin Infect Dis.

[8] Svraka S, Duizer E, Vennema H, de Bruin E, van der Veer B, Dorresteijn B, Koopmans M (2007). Etiological role of viruses in outbreaks of acute gastroenteritis in the Netherlands from 1994 through 2005. J Clin Microbiol.

[9] Lee LE, Cebelinski EA, Fuller C, Keene WE, Smith K, Vinjé J, Besser JM (2012). Sapovirus outbreaks in long-term care facilities, Oregon and Minnesota, USA, 2002-2009. Emerg Infect Dis.

[10] Norovirus Working Party. Guidelines for the management of norovirus outbreaks in acute and community health and social care settings. London: Public Health England; 2012.

[11] Care Quality Commission. Registration under the Health and Social Care Act 2008: Guidance on statutory notifications. London: Care Quality Commission; 2015.

[12] Public Health England (2014). Management of Outbreaks of Diarrhoea and Vomiting in Care Homes - North East Resource Pack.

[13] Kara-Zaïtri C, Gelletlie R, Schweiger M (2012). The development and deployment of a national web-based system for communicable disease control in England. Int J Infect Dis.

[14] United Kingdom Department of Health. Updated Guidance on the Diagnosis and Reporting of *Clostridium difficile*. London: United Kingdom Department of Health; 2012.

[15] Public Health England Standards Unit. Investigation of Faecal specimens for enteric pathogens. In: UK Standards for Microbiology Investigations. United Kingdom: Public Health England; 2014.

[16] Heinze G, Wallisch C, Dunkler D (2018). Variable selection - a review and recommendations for the practicing statistician. Biom J.

[17] Zeileis A, Hothorn T (2002). Diagnostic checking in regression relationships. R News.

[18] R Core Team (2018). R: a language and environment for statistical computing.

[19] Petrignani M, van Beek J, Borsboom G, Richardus JH, Koopmans M (2015). Norovirus introduction routes into nursing homes and risk factors for spread: a systematic review and meta-analysis of observational studies. J Hosp Infect.

[20] Inns T, Keenan A, Huyton R, Harris J, Iturriza-Gomara M, O’Brien SJ, Vivancos R (2018). How timely closure can reduce outbreak duration: gastroenteritis in care homes in north West England, 2012–2016. BMC Public Health.

[21] Barret AS, Jourdan-da Silva N, Ambert-Balay K, Delmas G, Bone A, Thiolet JM, Vaillant V (2014). Surveillance for outbreaks of gastroenteritis in elderly long-term care facilities in France, November 2010 to May 2012. Eurosurveillance.

[22] Ryan MJ, Wall PG, Adak GK, Evans HS, Cowden JM (1997). Outbreaks of infectious intestinal disease in residential institutions in England and Wales 1992-1994. J infect.

[23] Greig JD, Lee MB (2009). Enteric outbreaks in long-term care facilities and recommendations for prevention: a review. Epidemiol Infect.

[24] Tam CC, Rodrigues LC, Viviani L, Dodds JP, Evans MR, Hunter PR, Gray JJ, Letley LH, Rait G, Tompkins DS (2012). Longitudinal study of infectious intestinal disease in the UK (IID2 study): incidence in the community and presenting to general practice. Gut.

[25] Kirk MD, Fullerton KE, Hall GV, Gregory J, Stafford R, Veitch MG, Becker N (2010). Surveillance for outbreaks of gastroenteritis in long-term care facilities, Australia, 2002–2008. Clin Infect Dis.

[26] Lopman BA, Reacher MH, Vipond IB, Sarangi J, Brown DWG (2004). Clinical manifestation of Norovirus gastroenteritis in health care settings. Clin Infect Dis.

[27] Inns T, Trindall A, Dunling-Hall S, Shankar AG (2016). Introduction of a new rotavirus vaccine: initial results of uptake and impact on laboratory confirmed cases in Anglia and Essex, United Kingdom, July 2015. Hum Vaccin Immunother.

[28] Public Health England. PHE National norovirus and rotavirus Report. London: Public Health England; 2019.

[29] Inns T, Clough HE, Harris JP, Vivancos R, Adams N, O’Brien SJ (2019). Estimating the burden of care home gastroenteritis outbreaks in England, 2014–2016. BMC Infect Dis.

[30] Lynch M, Painter J, Woodruff R, Braden C (2006). Surveillance for Foodborne-disease Outbreaks: United States, 1998--2002. MMWR Surveill Summ.

[31] Stone ND, Ashraf MS, Calder J, Crnich CJ, Crossley K, Drinka PJ, Gould CV, Juthani-Mehta M, Lautenbach E, Loeb M (2012). Surveillance definitions of infections in long-term care facilities: revisiting the McGeer criteria. Infect Control Hosp Epidemiol.

[32] Rosenthal NA, Lee LE, Vermeulen BAJ, Hedberg K, Keene WE, Widdowson MA, Cieslak PR, VinjÉ J (2011). Epidemiological and genetic characteristics of norovirus outbreaks in long-term care facilities, 2003–2006. Epidemiol Infect.

[33] Ahmed SM, Hall AJ, Robinson AE, Verhoef L, Premkumar P, Parashar UD, Koopmans M, Lopman BA (2014). Global prevalence of norovirus in cases of gastroenteritis: a systematic review and meta-analysis. Lancet Infect Dis.

